# Effects of experimental drought and plant diversity on multifunctionality of a model system for crop rotation

**DOI:** 10.1038/s41598-024-60233-0

**Published:** 2024-05-04

**Authors:** Guylain Grange, Caroline Brophy, Rishabh Vishwakarma, John A. Finn

**Affiliations:** 1 Environment, Soils and Land Use Department, Teagasc, Johnstown Castle, Wexford, Y35 TC97 Ireland; 2 School of Computer Science and Statistics, Trinity College Dublin, Dublin 2, Ireland

**Keywords:** Ecology, Agroecology, Community ecology, Ecosystem services, Grassland ecology

## Abstract

In low-diversity productive grasslands, modest changes to plant diversity (richness, composition and relative abundance) may affect multiple ecosystem functions (multifunctionality), including yield. Despite the economic importance of productive grasslands, effects of plant diversity and environmental disturbance on multifunctionality are very rarely quantified. We systematically varied species richness, composition, and relative abundance of grassland ley communities and manipulated water supply (rainfed and drought) to quantify effects of diversity and environmental disturbance on multifunctionality. We then replaced the grassland leys with a monoculture crop to investigate ‘follow-on’ effects. We measured six agronomy-related ecosystem functions across one or both phases: yield, yield consistency, digestibility and weed suppression (grassland ley phase), legacy effect (effect on follow-on crop yield), and nitrogen fertiliser efficiency (full rotation). Drought reduced most ecosystem functions, although effects were species- and function-specific. Increased plant diversity affected mean performance, and reduced variation, across the six functions (contributing to multifunctional stability). Multifunctionality index values across a wide range of mixture diversity were higher than the best monoculture under both rainfed and drought conditions (transgressive over-performance). Higher-diversity, lower-nitrogen (150N) mixtures had higher multifunctionality than a low-diversity, higher-nitrogen (300N) grass monoculture. Plant diversity in productive grasslands is a practical farm-scale management action to mitigate drought impacts and enhance multifunctionality of grassland-crop rotation systems.

## Introduction

Agricultural systems can simultaneously affect the provision of several ecosystem functions that translate into services such as provision of food and fibre, maintenance of soil fertility, pest regulation, water purification, nutrient cycling etc.^1^ Narrowing the objectives of agricultural production systems to the sole aim of yield production may threaten the delivery of other crucial ecosystem functions. In grasslands, higher plant diversity may be needed to simultaneously support desired levels of multiple ecosystem functions and services^2^ and evidence supports this in semi-natural grasslands^3–5^. Productive agricultural grasslands in temperate areas typically rely on one or a low number of species with high yielding potential and good response to high and regular levels of fertiliser input and water supply. Although plant diversity is often equated to species richness, changing the identities and relative abundances of species in a community (i.e. changing community composition and evenness respectively) can also be influential. There remains significant knowledge gaps (but see Refs.^6–9^) about the role of changing plant species diversity as a practical farm-scale measure to improve simultaneous delivery of multiple ecosystem functions and services (multifunctionality) in productive grasslands.

Practical adaptation strategies are required to reduce the impact of climate change and drought events on grasslands. Climate change poses multiple challenges for the performance, sustainability and resilience of current and future farming systems^10^. Climate change effects will manifest in several ways that include changes in both mean and variability of temperature and precipitation, resulting in higher probability of extreme weather events (e.g. drought, heatwaves and flooding). Such weather effects result in economic damage^11,12^ and within grasslands, the impact of extreme weather events increases with management intensity^13,14^.

Species diversity in productive grassland mixtures can improve the delivery of selected ecosystem functions. Such mixtures comprise agronomic cultivars of grasses, legumes and herbs that are selected for high yields and digestibility by livestock; in addition, mixture benefits are strongly related to the contribution of legume species. The positive effects of plant diversity on agronomic responses include: yields^15^, weed suppression^16^ and total nitrogen yield^17^; no negative effects on forage quality^18^; positive effects on nitrous oxide emissions intensity^19^, and; improved resistance and resilience of grasslands to drought^20^. In general, such studies on productive grasslands conducted univariate analysis of single functions and there has been relatively few studies of multifunctionality. Different approaches to the analysis of multifunctionality are possible. Across a range of ecosystem types, several approaches analyse species richness as the main driver of multifunctionality^2,4,21,22^; however, these do not incorporate other important aspects of diversity e.g. composition and relative abundance of species within a level of species richness. In contrast, Diversity-Interactions modelling is a regression-based framework that was developed to model the univariate^23,24^ and multivariate^8^ BEF relationship; it is a more nuanced assessment of species diversity effects that is based on a broader definition of species diversity than richness alone^6,8,25^ and uses a combination of species composition (identity), richness and relative abundance. The manipulation of such diversity parameters is easily achieved within productive grasslands, and is a focus for targeted selection of best-performing combinations^24,26^. The Diversity-Interactions model for the multifunctional BEF relationship^8^ implicitly allows for association among the ecosystem functions to be quantified via the variance–covariance structure of the model error term. Despite some examples^6–9^, the amount of experimental research on multifunctionality in productive grasslands is surprisingly low, given the economic importance of this system, its provision and reliance on multiple ecosystem functions and services, and its exposure to the effects of climate change.

There is increasing attention on the design of arable crop production systems to improve soil fertility, nutrient use efficiency and crop yields, and to reduce negative environmental effects. There is considerable focus on the optimum way to diversify these highly specialised systems, and one prominent strategy is the use of multispecies grassland leys as part of a crop rotation. Grassland leys have the potential to increase soil carbon and the transfer of nutrients from the ley to the follow-on cereal crop (‘legacy effects’); they can also disrupt weed, pest and disease life cycles. Experiments have rarely investigated the role of grassland diversity in regulating legacy effects from the preceding grassland ley to the subsequent arable crop. However, experimental manipulation of grassland richness and relative abundance found either strong effects of grass-legume interactions (Fox et al.^27^) or of legume proportion only (Grange et al.^28^). There is, therefore, strong potential for the design of grassland leys to positively affect a crop rotation; however, quantitative analyses need to better reflect (1) the effect of plant diversity in species-poor systems on not just yield, but other ecosystem functions and services, (2) the combined effects across different phases of a crop rotation, to best assess the overall effect, and (3) the relative effect of environmental disturbance compared to the effects of plant diversity. Given the expected increase in environmental disturbance due to chronic and acute effects of climate change, food security can be better informed by knowledge of the effects of plant diversity in mitigating and adapting agroecosystems to environmental disturbance.

Here, we conduct a multifunctionality analysis on data arising from a two-phase model crop rotation experiment. Grange et al.^29^ showed benefits of mixing species for yield and the mitigation of drought effects across two years of a grassland ley. Grange et al.^28^ showed the strong effect of sown legume proportion in the same grassland ley phase on legacy effects in the follow-on crop. In the grassland ley stage, we manipulated plant diversity using six species from three functional groups (grasses, legumes and herbs) and imposed a water supply treatment with two levels (rainfed and drought), all managed at a low level of nitrogen application. We also included a replicated high nitrogen grass monoculture (*Lolium perenne*) treatment; this low-diversity, high-nitrogen community serves as a ‘control’ reference as it is widely used in practice in temperate regions. Here, we assess the multivariate relationship on multiple functions collected across our crop rotation experiment by fitting a multivariate Diversity-Interactions model^8^. We analysed six ecosystem functions across one or both phases: yield, yield consistency, digestibility and weed suppression (grassland ley phase), the legacy effect (yield of the crop phase), and nitrogen fertiliser efficiency (full two-phase rotation). Although we refer to our work as a multifunctionality analysis, our ecosystem functions studied are primarily related to agronomic production, stability and quality. The individual ecosystem functions we studied are not necessarily independent, and trade-offs can occur. We disentangle the contribution of each species and interspecific interactions between species or functional groups across two water supply treatments, and their effects on six agronomic ecosystem functions. We address the following questions:

- How does ley plant diversity (the number, identities and relative abundances of species) affect the multi-functionality of a crop rotation and how is the relationship altered by experimental drought conditions?
- How do individual functions perform in crop rotations across ley plant diversity and water supply gradients, and are there trade-offs in the context of multifunctionality?
- How does the multifunctionality of high-diversity, low-nitrogen communities compare to that of a low-diversity, high-nitrogen community, and is the comparison affected by drought?

## Results

### Effects of plant diversity and drought on individual functions

**Figure 1:**
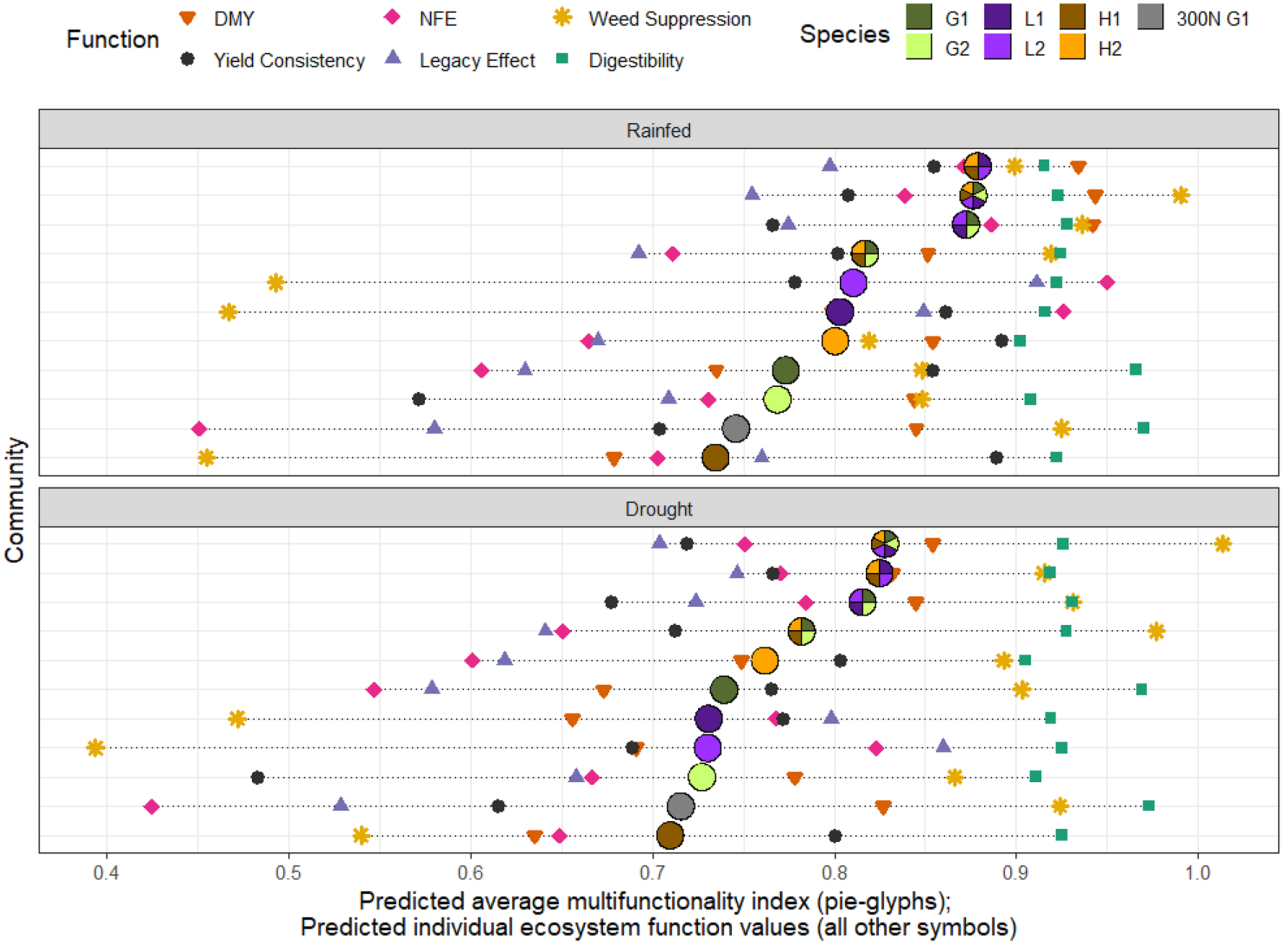
The predicted individual ecosystem functions (symbols) and predicted multifunctionality index (pie-glyphs) for a range of communities for rainfed and drought conditions. The sown proportions of each community are shown in the pie-glyphs for the MF index; G1 = *L*. *perenne*, G2 = *P*. *pratense*, L1 = *T*. *pratense*, L2 = *T*. *repens*, H1 = *C*. *intybus* and H2 = *P*. *lanceolata*, 300N G1 = the high N *L. perenne* monoculture comparison. The horizontal dotted lines highlight the range of individual predictions for each selected community. The communities are ordered according to the rank of their predicted multifunctionality index.

Parameter estimates of fixed effects from the final model are displayed in Table 1, and the variance covariance matrix estimates are displayed in Supplementary Table S1. For most functions, there was variation across the estimates of species’ identity effects (Table 1, Fig. 1, Supplementary Fig. S1). These species-specific effects generally indicated that *P. lanceolata* and *P. pratense* improved dry matter yield (DMY); both grass species and *P. lanceolata* promoted weed suppression; legume species were associated with higher legacy effects and nitrogen fertiliser efficiency (NFE); herb species encouraged better yield consistency, and; all species performed well for digestibility. Three of the six functions showed strong and positive effects of interspecific interactions that were related to functional group (FG) diversity. For these functions (DMY, NFE and weed suppression), the between-FG interactions (Grass*Legume, Grass*Herb, and Legume*Herb) were always significant and positive (Table 1). Within-FG interactions (Grass*Grass, Legume*Legume and Herb*Herb) were generally smaller, but always positive when significant (Table 1). The three other functions (yield consistency, legacy effect, and digestibility) were best estimated from species identities (Table 1), as there was no evidence of interspecific interactions for these functions.

**Table 1:** Estimates of fixed effects from the final multivariate Diversity-Interactions model (Eqs. 2a and 2b). Interaction terms and drought (additive) effects are in bold when significantly different from zero (α = 0.05).

	DMY		NFE		Weed suppression		Yield consistency		Legacy effect		Digestibility		MF index	
	Rainfed	Drought	Rainfed	Drought	Rainfed	Drought	Rainfed	Drought	Rainfed	Drought	Rainfed	Drought	Rainfed	Drought
(a) Fixed Effects														
Species identity														
* L. perenne*	0.735	0.673*	0.606	0.547*	0.848	0.903	0.854		0.630		0.966		*0.773*	0.739***
* P. pratense*	0.844	0.778***	0.731	0.667**	0.848	0.866	0.572		0.709		0.908		*0.769*	0.727***
* T. pratense*	0.799	0.656***	0.926	0.768***	0.467	0.472	0.861		0.849		0.916		*0.803*	0.731***
* T. repens*	0.808	0.691***	0.950	0.823***	0.493	0.394***	0.778		0.911		0.922		*0.811*	0.731***
* C. intybus*	0.679	0.635*	0.703	0.649*	0.455	0.540**	0.889		0.760		0.922		*0.735*	0.710*
* P. lanceolata*	0.854	0.749***	0.665	0.601**	0.819	0.893**	0.892		0.670		0.902		*0.801*	0.762***
Interaction														
Grass*Grass	0.110		0.029		− 0.007								* 0.022*	
Legume*Legume	− 0.016		** 0.205**		0.086								*** 0.071***	
Herb*Herb	0.135		− 0.086		** 0.476**								* 0.062*	
Grass*Legume	** 0.519**		** 0.275**		** 1.069**								*** 0.310***	
Grass*Herb	** 0.270**		** 0.154**		** 0.588**								*** 0.169***	
Legume*Herb	** 0.566**		** 0.211**		** 1.221**								*** 0.333***	
300N *L. perenne*	0.845	0.827	0.451	0.425	0.925	0.924	0.704		0.580		0.970		*0.746*	0.716**
Drought (Additive)								− **0.089**		− **0.051**		0.003		
(b) Selected communities														
Monoculture average	0.786	0.697***	0.763	0.676***	0.655	0.678	0.808	0.729***	0.755	0.704***	0.923	0.925	0.782	0.734***
2/3 L. perenne + 1/3 T. repens	0.875	0.794***	0.781	0.700***	0.967	0.971	0.829	0.740***	0.724	0.673***	0.951	0.954	0.855	0.805***
6-species equi-proportional mixture	0.943	0.854***	0.838	0.751***	0.99	1.013	0.808	0.729***	0.755	0.704***	0.923	0.925	0.877	0.828***

Differences between rainfed and drought estimates for species identity effects and 300N *L. perenne* are indicated as ***P ≤ 0.001, **P ≤ 0.01, *P ≤ 0.05 for dry matter yield, nitrogen fertiliser efficiency and weed suppression, each of which included an interaction with the water supply treatment for these terms. For yield consist., legacy effect and digestibility, the difference between rainfed and drought was a constant additive effect (NS for digestibility). The final column ‘MF index’ was predicted from the multivariate Diversity-Interactions model (see “Methods”). (b) Predicted values from the final model for selected communities for each function individually and for the MF index. Differences between rainfed and drought predictions are indicated by stars; this inference via the multivariate Diversity-Interactions model respects the multivariate nature of ecosystem multifunctionality. Ecosystem function notation: *DMY* dry matter yield, *NFE* nitrogen fertiliser efficiency, *MF index* multifunctionality index.

Except for digestibility, the drought treatment caused a significant reduction in each ecosystem function compared to the rainfed treatment. For DMY, NFE and weed suppression, the drought effect was species-specific (cf. the species’ identity effects under rainfed and drought in Table 1). Drought had no impact on the 300N community performance for DMY, NFE and weed suppression (Table 1). For yield consistency and the legacy effect, drought had a negative impact that was constant across all sown community types, while drought had no effect on digestibility (Table 1).

### No plant community was optimal for all six functions under rainfed and drought conditions

No monoculture promoted high levels of performance across all six functions. All monocultures had low performance in at least one function and water supply level; the lowest-performing function was NFE for 300N *L*. *perenne* and *P*. *lanceolata*, yield consistency for *P*. *pratense*, and weed suppression for both legume species and *C*. *intybus* (Fig. 1). Note also the considerable change in magnitude and rank order of the six functions across the monoculture communities, which is indicative of trade-offs among monocultures in the delivery of different ecosystem functions. Under each water supply level, 300N *L. perenne* performed poorly in both the NFE and the legacy effect (Table 1, Fig. 1). For the 300N treatment, yield consistency and the legacy effect were reduced under drought, but drought did not affect any of the other ecosystem functions for this community. The only ecosystem function with high performance across all communities was digestibility—it was quite high and stable across both species diversity and water supply levels (Fig. 1).

**Figure 2:**
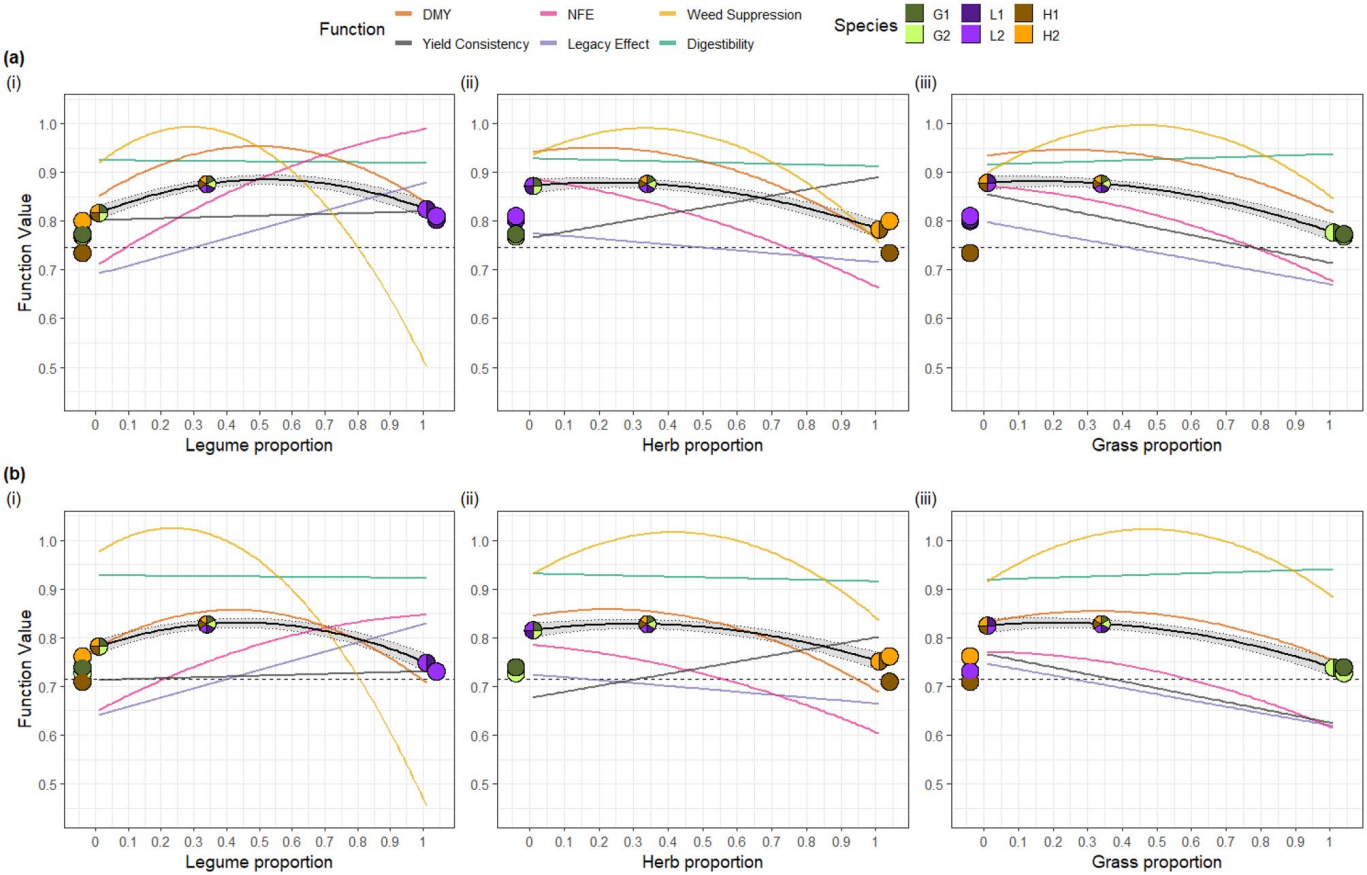
The predicted individual ecosystem functions (coloured lines) and predicted multifunctionality index (black line, with 95% confidence bands based on inference from the multivariate Diversity-Interactions model) versus sown proportion of (i) legume, (ii) herb and (iii) grass functional groups (assuming equal proportions for the two species within each functional group, and equal proportions of each of the two functional groups not being highlighted within the panel) for (**a**) rainfed and (**b**) drought conditions. Predictions highlighted by pie-glyphs are for the MF index, with the sown proportions of each community illustrated; G1 = *L*. *perenne*, G2 = *P*. *pratense*, L1 = *T*. *pratense*, L2 = *T*. *repens*, H1 = *C*. *intybus* and H2 = *P*. *lanceolata*. For reference, the predicted MF index for the 300N *L. perenne* monoculture comparison is included as a horizontal dashed line (but is not related to the x-axis gradient).

Although plant diversity generally improved performance (e.g. Supplementary Fig. S2), no mixture community optimised performance across all six individual functions. Mixtures that omitted legume species (e.g., the four-species mixture of the two grass species and the two herb species), had reduced performance in NFE and the legacy effect, compared to mixtures that included legumes (Fig. 1, cf. the four-species mixture of the two grasses and two herbs with the three other mixtures, p < 0.001 for each comparison for both NFE and the legacy effect; Supplementary Fig. S2); thus, including legume species in a mixture improved multifunctionality. As the sown legume proportion changed, the equi-proportional six-species mixture (legume proportion ≈ 0.33) was close to the optimal for weed suppression, DMY and digestibility, while increasing the sown legume proportion above 0.33 improved the legacy effect and NFE, but strongly reduced weed suppression (Fig. 2i). Similarly, as the herb proportion (Fig. 2ii) or grass (Fig. 2iii) proportion were increased beyond ≈ 0.33, trade-offs occurred as no one plant community simultaneously optimised all six functions (Fig. 2).

### Higher and more stable ecosystem multifunctionality in more diverse multi-species mixtures than monocultures under both rainfed and drought conditions in a model crop rotation

**Figure 3:**
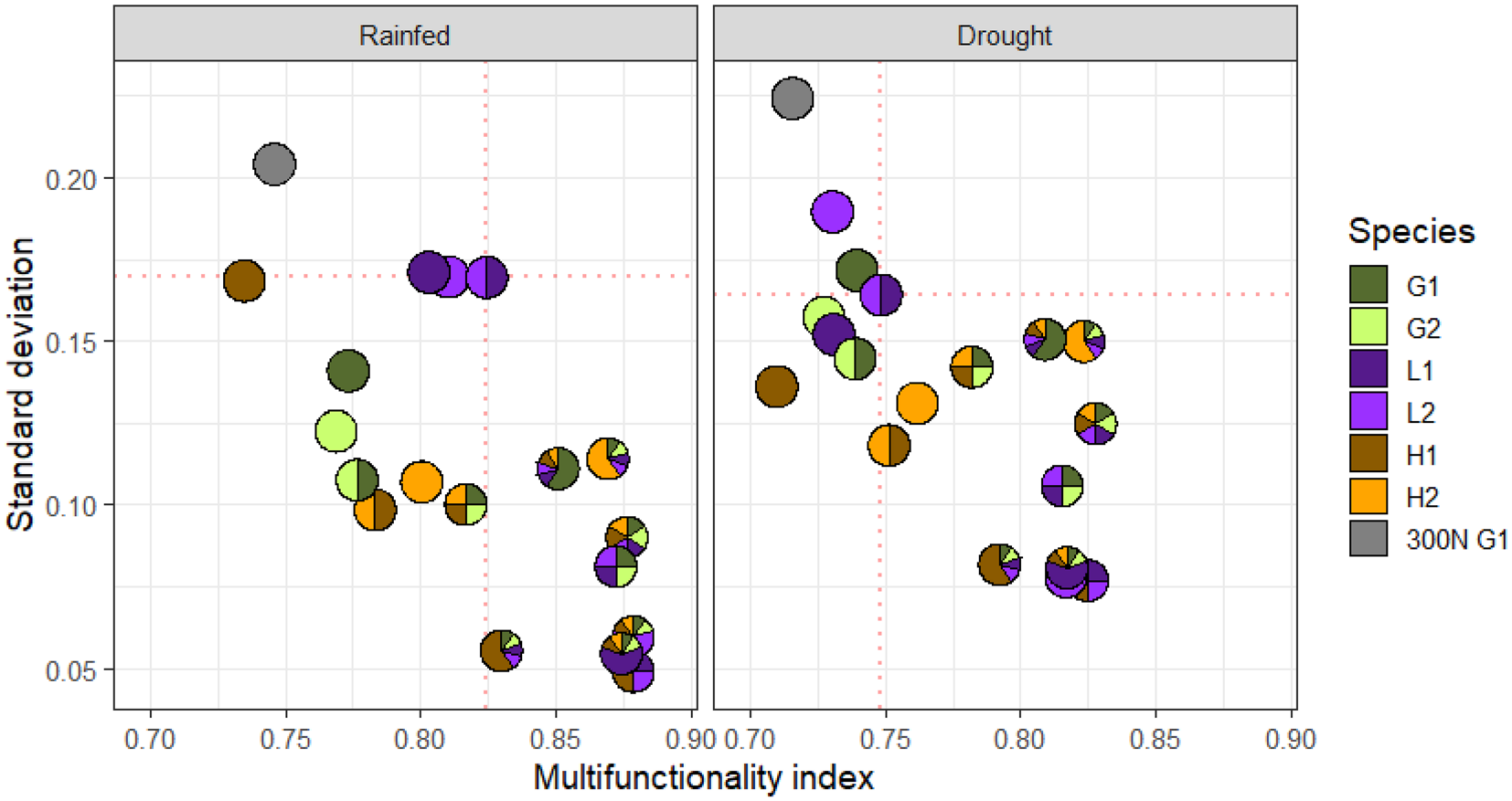
The standard deviation versus the predicted multifunctionality (MF) index for a range of communities (from our experimental design) for rainfed (left panel) and drought (right panel) conditions. The MF index was predicted for each community from the multivariate Diversity-Interactions model (Eq. 2). The ‘standard deviation’ for each community was computed as the standard deviation across the six individual ecosystem function predictions from the multivariate Diversity-Interactions model. The sown proportions of species in each community are shown in the pie glyphs; G1 represents *L*. *perenne*, G2 = *P*. *pratense*, L1 = *T*. *pratense*, L2 = *T*. *repens*, H1 = *C*. *intybus* and H2 = *P*. *lanceolata*, 300N G1 represents the 300N *L. perenne* comparison. The dotted lines are through the centre of the prediction for the mixture community closest to the top left corner, included for visual reference. For maximal provision of the ecosystem functions, the ideal location for communities is near the bottom right-hand corner (higher MF index that is more stable across functions).

Compared to monocultures, the MF index was higher for communities that mixed two or three functional groups, and the standard deviation across the six ecosystem functions for these mixtures was lower, even for mixture communities in which one of three functional groups was dominant (Fig. 3). In both rainfed and drought conditions (Fig. 3), monocultures are located towards the top left corner (lower mean, higher variation), whereas mixtures of four, five or six species are towards the bottom right corner (higher mean, lower variation). In general, the multifunctionality index was a little lower and standard deviation a little higher under drought compared to rainfed conditions. The 300N *L. perenne* community had the highest standard deviation under both water supply treatments and was among the lowest-performing communities for the multifunctionality index.

### Transgressive overperformance in multifunctionality index was strongly related to mixture diversity and generally reduced by drought

**Figure 4:**
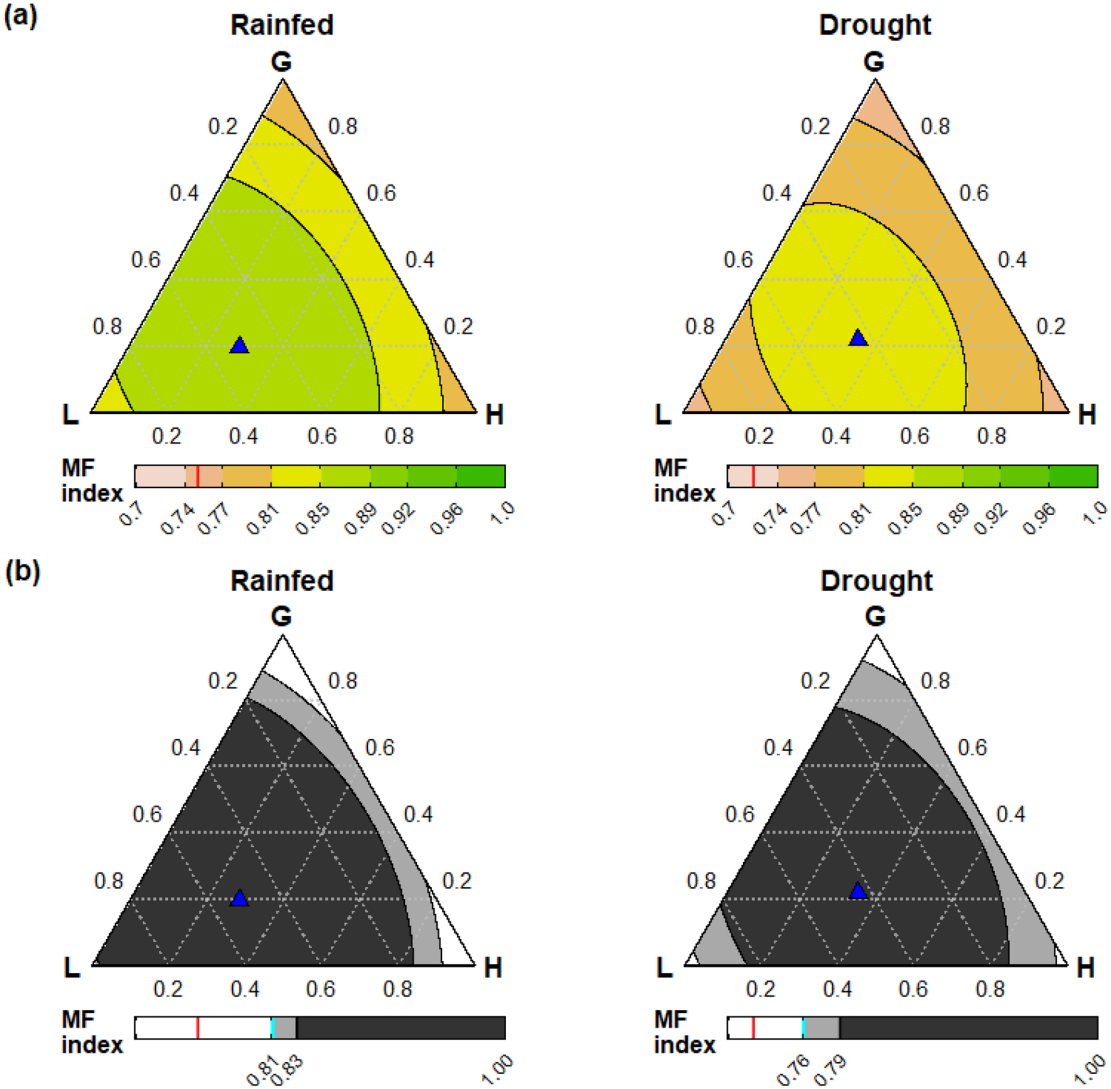
(**a**) Contour plots showing predicted values (from the multivariate Diversity-Interactions model estimates in Table 1) of the multifunctionality (MF) index from any mixture sown with grass, legume and/or herb under rainfed (left) or drought (right) conditions, assuming equal proportions for the two species within each functional group. Values of the MF index were predicted across the functional group simplex space for two species (both from one functional group—the vertices in each ternary diagram), four species (with two species from each of two functional groups—the sides of each ternary diagram) or all six species (three functional groups—all interior points in the ternary diagrams). The blue triangle indicates the community with the maximum performance, and the red bar in the colour legend displays the predicted value for the 300N *L. perenne* monoculture. Note that MF predictions for monocultures of individual species are not shown in this plot (but are shown in Fig. 3). (**b**) Contour plots with the MF index of mixtures in comparison to the best-performing monoculture (*T. repens* under rainfed and *P. lanceolata* under drought, displayed with a cyan-coloured bar on the colour legend in each panel). The white area represents mixture communities with a MF index value that is less than that of the best monoculture; the light grey area represents mixtures with MF index value > best monoculture, and the black area represents mixtures with MF index values significantly greater than the best monoculture (at the $\alpha =0.05$ level), i.e., significant transgressive over-performance.

The multifunctionality index was strongly affected by plant diversity (the MF index is predicted from the final Diversity-Interactions model, estimates shown in Table 1 and displayed in Fig. 4a); the magnitude of the benefit from species interactions for the MF index (driven by the three ecosystem functions with synergistic species interactions, Table 1) was strong enough to result in transgressive over-performance across a wide range of mixtures, i.e. mixtures outperformed the best-performing monoculture (Fig. 4b). Higher MF index values were achieved by more diverse communities (i.e., around the central area of the ternary diagram), and displayed considerable robustness to change in relative abundance around the equi-proportional mixture of three functional groups i.e., a large area in the ternary diagram has MF index value greater than, for example, 0.85 in the rainfed treatment. In particular, communities sown with 20–80% legume delivered a higher MF index than the best monoculture under both rainfed and drought conditions (Fig. 4b), i.e., transgressive over-performance in the multifunctionality index. The predicted performance of a mixture of 67% *L. perenne* and 33% *T. repens* had a significantly higher MF index than any monoculture, under both drought and rainfed conditions (Table 1b). It, in turn, was significantly outperformed by the six-species equi-proportional mixture (P = 0.002) in both rainfed and drought conditions.

The MF index value of the 300N *L. perenne* monoculture was below the range of predicted performance of mixtures shown in the ternary diagrams, including the vertices where only one FG is represented (Fig. 4a). The 300N *L. perenne* monoculture MF index prediction of 0.75 under rainfed conditions was outperformed by the vast majority of 150N mixtures, including those under drought conditions.

## Discussion

We investigated six ecosystem functions within a model system of crop rotation comprising an intensively-managed grassland ley (that manipulated water supply (environmental disturbance) and plant diversity) and a follow-on monoculture crop. We used the multivariate DI modelling framework for analysis and visualisation of multifunctionality, advantages of which include: the retention of information on single functions as well as the summary of functions via the MF index; statistical inference that respects and utilises the multivariate nature of multifunctionality; quantification of how species and interspecific interactions affect all responses, and; quantitative prediction of how each function (and the MF index) performs across a range of species compositions, relative abundances and richness level (Dooley et al.^8^). For our site conditions and these species, there was substantial variation in the provision of the studied ecosystem functions across the different plant communities, and drought effects were species- and function-specific. Overall, more diverse multi-species mixtures (compared to monocultures or mixes of two species from the same FG) were associated with higher mean levels and lower variation in multifunctionality and were robust across a range of different relative abundance of the three FGs, providing evidence of multifunctional stability. The MF index value of the 300N *L. perenne* monoculture was lower than that of the vast majority of 150N mixtures.

### Diversity increased multifunctionality but did not maximise all functions

No single community maximised all studied functions. Among the monocultures, there was clear evidence of trade-offs in performance among the functions (Figs. 1 and 2). No mixture community achieved highest levels of performance across all functions, and there were clear trade-offs among some functions as relative abundances of the functional groups changed (Fig. 2, and Supplementary Fig. S2). Thus, correlations among functions were not all strongly positive and higher diversity did not maximise all functions (see Byrnes et al.^21,30^). In a four-species grass-clover experiment, Suter et al.^6^ showed that no single community maximised performance across ten individual agronomic functions (Figs. 1,3 in Suter et al.^6^); synergistic grass-clover interactions enhanced multifunctionality across a wide range of legume proportions (Fig. 3 in Suter et al.^6^). They also showed that multifunctionality of low-nitrogen mixtures was at least as high as that in high-nitrogen grass monocultures^6^. With a similar design and the same species as in our study, Argens et al.^7^ investigated seven ecosystem functions (related to agronomy, biodiversity, plant physiology and soil nitrogen), and found that different species compositions promoted different individual functions; although multifunctionality increased with functional group and species richness, it was maximal for legume-herb combinations. Dooley et al.^8^ found higher performance and lower variability across three agronomic functions in four-species mixtures compared to monocultures; they found that trade-offs occurred mostly in monoculture communities. Savage et al.^9^ investigated five ecosystem services (including biodiversity- and soil-related services) and management factors in three plant communities and concluded that “a single management solution to maximise the delivery of all ecosystem services is unlikely to exist, as trade-offs also occurred.” Thus our finding that no single community maximised all ecosystem functions is in broad agreement with comparable studies^6,7,9^.

We found that diversity strongly affected multifunctionality; however, it was dependent on the combination of species, functional group and relative abundance. Overall, we found a low effect of increasing species richness from one to two species within each FG; a modest effect of combining species from the grass and herb FGs; a strong effect of combining two FGs when one of them comprised legumes, and; a saturating response of the MF index as diversity increased to mixtures comprising three FGs. Of course, these broad patterns reflect the comparison of balanced communities (an even distribution of relative abundance among the component species), but shifts in species relative abundances can also have large effects (Fig. 4, Box 1).

Not all functions and services are necessarily of equal importance^1,21,25,30,31^ and the weighting of individual functions can have a big effect on the MF index (see Box 1). Our calculation of the MF index assumed equal weighting among the six responses, but our model can be used to predict and visualise the effect of different relative proportions and weightings (Box 1, Supplementary Fig. S2).

Box 1. Average thresholds method: interactive visualisation The accompanying online interactive visualisation [https://dimodels.shinyapps.io/average_thresholds/] compares the performance of various communities across all six functions in the experiment (boxplots from Supplementary Fig. S3a). The multifunctionality index (MF index) is calculated by using the multivariate model to compute a weighted average of the performances across the six functions for a wide range of communities. The weights can be set to range from 0 (no importance) to 1 (high importance) using the corresponding sliders (Supplementary Fig. S3b). The weighted MF index is calculated for grass, legume, and/or herb functional group mixtures across the simplex space, under both rainfed and drought conditions. There is a default 50–50% split between each of the two species within a functional group, but this can be varied (sliders in Supplementary Fig. S3c). The coloured ternary diagrams show a contour map of the weighted MF index for the different communities (Fig. 4a, main text). The grayscale figures (Fig. 4b, main text) compare the weighted MF index of the mixtures to that of the best-performing monoculture for that weighted combination of the responses (transgressive over-performance; see online version). The optimal proportion of species within a particular functional group for the chosen weighted MF index is estimated by clicking the ‘Optimize’ button (Supplementary Fig. S3). This optimization is performed by calculating the weighted MF index for each combination of species proportions within all three functional groups and selecting the combination of species proportions which gives the best performance for the weighted MF.

### Drought reduced multifunctionality, but in a relatively consistent manner across the diversity gradient

Experimental investigation of environmental disturbances on multifunctionality is relatively rare^32,33^. Here, the negative effects of drought on individual functions were largely manifested through effects on species (identity effects) and did not affect the interspecific interactions (being the same under drought as in the rainfed control). Overall, the broad pattern of the positive effect of diversity on multifunctionality was relatively consistent across the rainfed and drought treatments, and there were only modest changes in the rank order of the selected communities and individual functions between the rainfed and drought treatments (Figs. 1, 2, 3).

The requirement of higher diversity to maintain functioning across environmental gradients in space and/or time is well established^2,34^. A meta-analysis of 46 experiments showed that biodiversity effects on different ecosystem functions were larger in more stressful conditions and driven by interspecific complementarity^35^. Our experimental manipulation of water supply to create an environmental disturbance aligns with these conclusions; there was a strong response of the MF index to diversity under drought due to strong between-FG interactions, the magnitude of which was maintained under drought, despite species’ identity effects being lower under drought (Table 1 and Fig. 4a). Here, the maintenance of interaction effects under drought underpins the robustness of the BEF relationship e.g. the equi-proportional six-species mixture maintained the magnitude of its benefit (+ 0.095, Table 1 (b)) over the monoculture average under both drought and rainfed conditions.

Comparing the average of the six monocultures and the equi-proportional six-species mixture (Table 1), the negative effect of drought (− 0.048) on the MF index was lower than the positive effect of diversity (+ 0.095). For individual functions, only DMY and weed suppression showed greater positive effects of diversity than negative effects of drought, and this was sufficient for the MF index to respond more to diversity than drought. The MF index of the six-species equi-proportional mixture under drought outperformed or equalled that of the monocultures in rainfed conditions (Table 1), showing that mixtures offer an adaptation option for drought effects on multifunctionality of monocultures (see also Haughey et al.^36^).

### Interspecific interactions underpinned transgressive over-performance in MF index

A wide range of mixture communities (with varying relative abundance of the six species) outperformed the monoculture with the best MF index, but this depended on the composition and relative abundances of the functional groups (Fig. 4b). Transgressive over-performance is only possible due to synergistic interspecific interactions^23,37^, and the strongest interaction effects derived from mixing of species from different functional groups, compared to pairs of species within functional groups (Table 1). Importantly, equi-proportional mixtures of two species from the same FG (vertices in Fig. 4, Table 1) never significantly outperformed the best monoculture (due to insufficiently strong within-functional group interaction effects). However, under both rainfed and drought conditions, four-species mixtures with two FGs achieved transgressive over-performance in MF index if they included the legume FG and neither FG had < 20% sown proportion (see the edges in Fig. 4b). This shows the benefit of balanced communities with FG diversity to achieve high multifunctionality. This very strong joint influence of functional group composition and relative abundance again highlights the importance of using a metric of diversity that includes species (and sometimes FG) composition and proportions rather than just species richness^24,38^. Such balanced communities correspond to the central area of the simplex design (shown in the ternary diagram, Fig. 4), where the MF index response shows the robustness of high levels of ecosystem functioning across varying species proportions. This robustness can also be explored with the interactive tool described in Box 1.

### Specific combinations of species can optimise performance

Productive temperate grasslands are expected to reduce environmental footprint as well as deliver forage quantity and quality, and the better performance of targeted combinations needs to include both these aspects. Within the general effects of FG and species diversity, we can use our model to identify and compare targeted combinations of species and functional groups that deliver high levels of performance across the selected functions. This is important for farming practice, which can (and aims to) implement grassland communities that maximise performance^26^ (Finn et al., accepted). For example, balanced four-species mixtures with the legume FG and one other FG achieved transgressive over-performance in MF index; even within this range, however, some targeted combinations of species may be better than others. Lower-diversity, higher-input management (exemplified here by 300N *L. perenne*) was associated with lowest performance of NFE, yield consistency and the legacy effect. In contrast, the high-diversity, low-input management (e.g. 150N six-species equi-proportional mixture) delivered high (but not necessarily maximal) levels of all six functions. There was higher (P < 0.001) multifunctionality in higher-diversity, lower-input grassland communities (MF index = 0.877 in equi-proportional six-species mixtures, rainfed conditions; MF index = 0.828, drought conditions)) than in the low-diversity, high-input 300N *L. perenne* community (MF index = 0.746, rainfed conditions) (Table 1, and see dashed lines in Fig. 2). A monoculture of *L. perenne* with 300N had significantly lower MF index values than the six-species grass-legume-herb mixture with 150N. Thus, we show higher multifunctionality in higher-diversity, lower-input grassland communities than from a low-diversity, high-input community. Comparing across a wider suite of targeted combinations that varied in nitrogen fertiliser input and plant diversity, each of the following communities (rainfed treatment) had a significantly (P < 0.05) lower MF index value than the next in the list: 300N *L. perenne* monoculture < 150N *L. perenne* monoculture < 150N best monoculture < 150N two-species mixture of 2/3 *L. perenne* and 1/3 *T. repens* < 150N six-species equi-proportional mixture. Future investigations could further quantify the generality of the added value of increasing plant diversity in productive grasslands beyond the two-species combination of perennial ryegrass and white clover widely implemented in productive temperate grasslands^39^. Quantification of the effect of plant diversity on multifunctionality in ley-crop agroecosystems would benefit from longer temporal scales and a wider set of environmental conditions through multi-site experiments. Such experiments would improve generality of interpretation, and also better assess the degree and extent of higher mean levels and lower variation in MF index, which reflects multifunctional stability (within the drought treatment). Overall, the capacity of plant diversity to deliver multiple functions and services within agro-ecosystems can be a practical, farm-scale management option to improve agricultural sustainability as well as mitigate and adapt to environmental disturbance.

## Methods

### The field experiment

**Figure 5:**
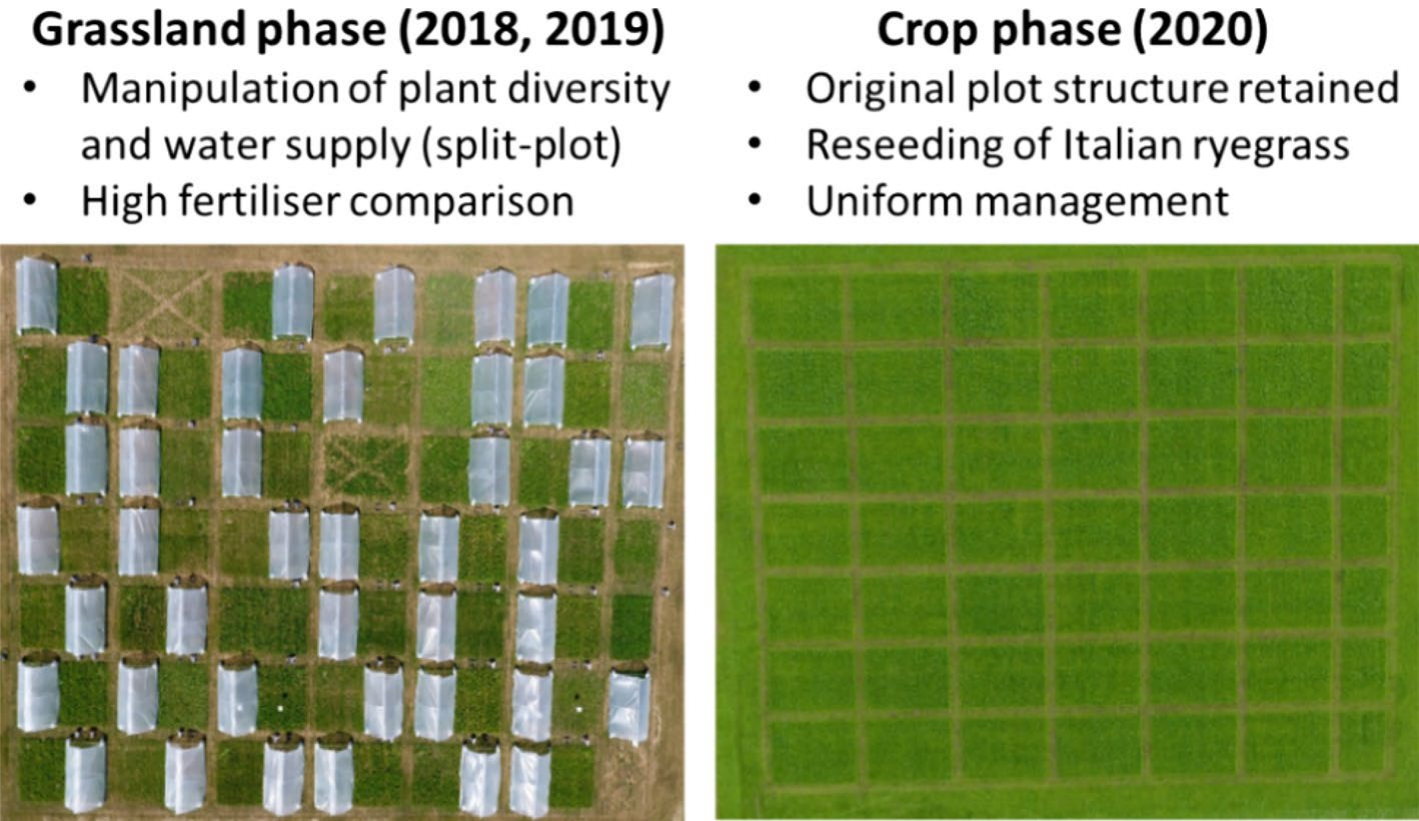
Field layout and main characteristics for both the grassland and following crop phase of the experiment. See text for details of the six functions measured (adapted from Grange et al.^29^).

A three-year grassland-crop rotation experiment was carried out in Johnstown Castle, Co. Wexford, in the south-east of Ireland. The grassland phase of the rotation was established in April 2017 and plots were measured for two years following the year of sowing (2018 and 2019^29^). The follow-on crop phase commenced in spring of 2020^28^ and plots were measured over the growing season in that year. An overview of the crop rotation experiment and the measurements collected in the two phases is in Fig. 5.

In the grassland phase, plots of size 5 m × 7 m were sown with varying combinations of plant species using a pool of six species from three functional groups: species 1 and 2 were grasses (*Lolium perenne* and *Phleum pratense*), species 3 and 4 were legumes (*Trifolium pratense* and *Trifolium repens*) and species 5 and 6 were herbs (*Cichorium intybus* and *Plantago lanceolata*). These plant species are neither at risk of extinction nor threatened, and their use complied with relevant institutional, national, and international guidelines and legislation. Following a simplex design, 19 plant communities were systematically assembled in a total of 39 plots, ranging from monocultures to six-species mixtures with sown proportions of each species at 0%, 10%, 25%, 50%, 60% and 100% depending on the community (full design in Table S2). In this phase, all plots were fertilised with an annual rate of 150 kg of nitrogen (N) (150N), 30 kg of phosphorus and 300 kg of potassium per hectare per year. Four additional plots of a *L. perenne* monoculture received double the amount of N (300N) as a higher-nitrogen reference, giving a total of 43 plots. To simulate the effect of a summer drought, each of the 43 plots (i.e. main plots) was divided into two 5 m × 3.5 m split plots and the subplots were randomly assigned to either a rainfed control or an experimental drought treatment. Drought was simulated by covering the drought subplots with rainout shelters for a two-month period in 2018 and 2019. Rainfall exclusion resulted in extreme drought stress for the two summers (soil water potential < − 1.5 MPa) (as also described in Grange et al.^29^).

During the grassland phase, there were seven annual harvests where total aboveground biomass was cut to 4.5 cm in height using a Haldrup harvester each time (14 harvests in total in this phase). At each harvest, dry matter yield was determined for each subplot after oven drying samples of the material harvested. The dry samples were then ground and sent for infrared spectroscopy analysis to assess nitrogen content and digestibility of the material^40^. For three harvests in each year, a representative sample of the freshly cut material was manually sorted to measure the proportion of plant biomass comprising each of the sown species and weeds.

In March 2020, the grassland phase was terminated by application of glyphosate. The plots were reseeded in April 2020 with a monoculture of *Lolium multiflorum* that served as a model crop, keeping the same experimental field layout of subplots/plots. All plots were managed uniformly in 2020 and both subplots within each plot were also treated identically, i.e. there was no manipulation of drought in the follow-on crop phase. All plots/subplots received the same fertiliser in this phase (40 kg N ha^−1^), including the plots that received the high N treatment in the grassland phase. Homogeneous management in this phase enabled isolation of the effects of the treatments in the grassland phase (plant diversity, drought and high N) on the follow-on crop performance. Each sub-plot was harvested on four occasions during 2020, and dry matter yield and nitrogen content were determined for each subplot at each harvest.

### Ecosystem functions measured

We analysed the following six ecosystem functions recorded on each subplot. From the grassland phase, we measured four functions: dry matter yield (DMY) was measured as the average annual yield from the two years of the grassland phase; the standard deviations (SD) of yield across the seven harvests in each year during the grassland phase were averaged and used as the indicator of yield consistency (1/SD); pepsin-cellulase digestibility was assessed using near infrared spectroscopy on the harvested forage material across the 14 grassland phase harvests (weighted by harvested biomass), and; weed proportion was averaged across the six harvests (three in each of two years that were sorted (weighted by harvested biomass) and weed suppression was calculated as (1—weed proportion). From the follow-on crop phase, the annual yield of the follow-on crop (summed across four harvests) indicated the legacy effect^27^ of the grassland ley on the follow-on crop of *L. multiflorum*. Across the full rotation, total nitrogen yield in all harvested forage material across both the grassland and crop phases was divided by the total amount of nitrogen fertiliser spread across both phases to calculate the overall nitrogen fertiliser efficiency (NFE).

### Data preparation

To enable simultaneous analysis of multiple functions, we standardized all measurements to a comparable scale^8^. The raw data for each function was standardized by dividing each variable by the average of its top three values. For most of the functions, high values corresponded to high achievement from an agronomic perspective (DMY, legacy effect, digestibility, NFE). Weed suppression was already on a scale of 0 to 1 and so was not standardised. For yield consistency, small standard deviation values are considered more desirable, thus, the three minimum values were used as a reference and the standardizing ratio was inverted (1/SD). The standardization process aimed to scale the data between 0 and 1, with value = 1 being the high achievement reference for each function. In practice, some values may lie a little above 1 (due to division by the average of the three highest values); the standardized values across all functions ranged from 0.36 to 1.05.

### Statistical modelling

We used the multivariate Diversity-Interactions model^8^ to quantify the effect of plant diversity and water supply on several ecosystem functions and incorporate the covariances between functions as part of the model. As a starting point for the model fitting process, the linear predictor for each ecosystem function (*k*) took the form:

1 $$\left(\sum_{i=1}^{6}{\beta }_{ik}{P}_{i} +\sum_{\begin{array}{c}i,j=1\\ i<j\end{array}}^{6}{\delta }_{ijk}{P}_{i}{P}_{j} +{\gamma }_{k}{X}_{N}\right)\times Treatment$$

where ${P}_{i}$ is the sown (in the grassland ley phase) proportion of species $i$ and the associated parameters are species identities ($\beta$ coefficients) and interspecific interactions ($\delta$ coefficients). A dummy variable for the high nitrogen comparison, ${X}_{N} = 1$ for 300N and 0 elsewhere, was included; when ${X}_{N}$ was equal to 1, all ${P}_{i}$ were set equal to 0, thus the $\gamma$ parameter is the expected value for the 300N *L. perenne* monocultures. In Eq. (1), the water supply treatment (rainfed or drought; denoted as a factor ‘*Treatment*’) was crossed with all other terms.

For a preliminary assessment of the form of the linear predictor (i.e., structure of identity, interaction, and water supply effects) for each ecosystem function, a range of univariate Diversity-Interactions models^23,24^ with varying fixed effects were fitted and compared separately for each function, using AICc (corrected AIC^41^) to compare models. In this assessment, a range of interspecific interaction terms were tested, including that all pairwise species interacted in the same way, and that species interactions were dictated by functional group membership. The extent to which the water supply treatment needed to be crossed with other model terms was also tested. These univariate models for each function informed the variables that would be included in the linear predictor for each ecosystem function in the multivariate Diversity-Interactions model. When fitting the multivariate Diversity-Interactions model, the error structure of the model allowed for both the split plot design and the multivariate nature of the response: (1) plot (i.e. main plot) was treated as random, with a normal distribution and a separate variance for each function to adjust for the split plot nature of the design; (2) the residual error term was assumed multivariate normally distributed and included a block diagonal structure with a block for each subplot that included a unique variance for each function and unique covariance between each pair of functions to capture the multivariate nature of the responses on each subplot.

### Selected multivariate Diversity-Interactions model

In preliminary analyses, the effect of species diversity and water supply treatment varied considerably across the six ecosystem functions analysed. The linear predictor for dry matter yield (DMY), nitrogen fertiliser efficiency (NFE) and weed suppression included species-specific identity effects and a term for the 300N comparison, both crossed with water supply treatment and interspecific interactions that were dictated by functional group membership (Eq. 2a). The linear predictor for yield consistency, the legacy effect and digestibility included species-specific identity effects, an additive effect of the water supply treatment and a term for the 300N comparison (Eq. 2b).

$$\left(\sum_{i=1}^{6}{\beta }_{ik}{P}_{i} +{\gamma }_{k}{X}_{N}\right)\times treatment+{\delta }_{GGk}{P}_{1}{P}_{2}+{\delta }_{LLk}{P}_{3}{P}_{4}+{\delta }_{HHk}{P}_{5}{P}_{6}$$

2a $$+{\delta }_{GLk}{P}_{G}{P}_{L}+{\delta }_{GHk}{P}_{G}{P}_{H}+{\delta }_{LHk}{P}_{L}{P}_{H}$$

2b $$\sum_{i=1}^{6}{\beta }_{ik}{P}_{i} +{\gamma }_{k}{X}_{N}+{\eta }_{k}{X}_{D}$$

${P}_{1}$ to ${P}_{6}$ are the sown proportions of species 1 to 6 respectively, ${P}_{G}={P}_{1}+{P}_{2}$ is the sown proportion of grass species, ${P}_{L}={P}_{3}+{P}_{4}$ is the sown proportion of legume species, while ${P}_{H}={P}_{5} +{P}_{6}$ is the sown proportion of herb species. In Eq. (2a), ‘treatment’ represents a water supply factor with two levels (rainfed and drought). In Eq. (2b), ${X}_{N}=0$ for 150 N and ${X}_{N}=1$ for 300 N, and ${X}_{D}=0$ for rainfed and ${X}_{D}=1$ for drought*.*

We used the final model in three ways to interpret the effects of the species diversity and water supply treatments on ecosystem multifunctionality. For a range of communities: (1) we predicted the multivariate (six-dimensional) ecosystem function responses from the model, (2) we predicted the mean of the six ecosystem functions, which we termed the multifunctionality index ‘MF Index’, and (3) we computed the uncertainty of the predictions across the six functions. Each of these was computed from our final multivariate Diversity-Interactions model. When predicting the MF Index from our multivariate Diversity-Interactions model, all functions were given equal weighting (but it is possible to adjust weightings in a scenario where some functions are deemed more important than others^3,25^). We used SAS/STAT software function ‘proc mixed’ for model fitting^42^ and predictions. Further information on interpreting Diversity-Interactions models is available in Kirwan et al.^23^ and Moral et al.^24^.

### Supplementary Information

Supplementary Information.

## Data Availability

Data, metadata and statistical code are available from Teagasc’s Open Access repository T-Stór at https://t-stor.teagasc.ie/handle/11019/3518.
