# Supplementary material for: Effects of experimental drought and plant diversity on multifunctionality of a model system for crop rotation
Source: Sci Rep. 2024 May 4;14:10265. doi: 10.1038/s41598-024-60233-0 (PMC11069507; doi:10.1038/s41598-024-60233-0)
Supplement: Supplementary file 1 — Supplementary Information. [file 41598_2024_60233_MOESM1_ESM.pdf]

Supplementary material

Grange et al. 'Effects of experimental drought and plant diversity on multifunctionality of a model system for crop rotation'

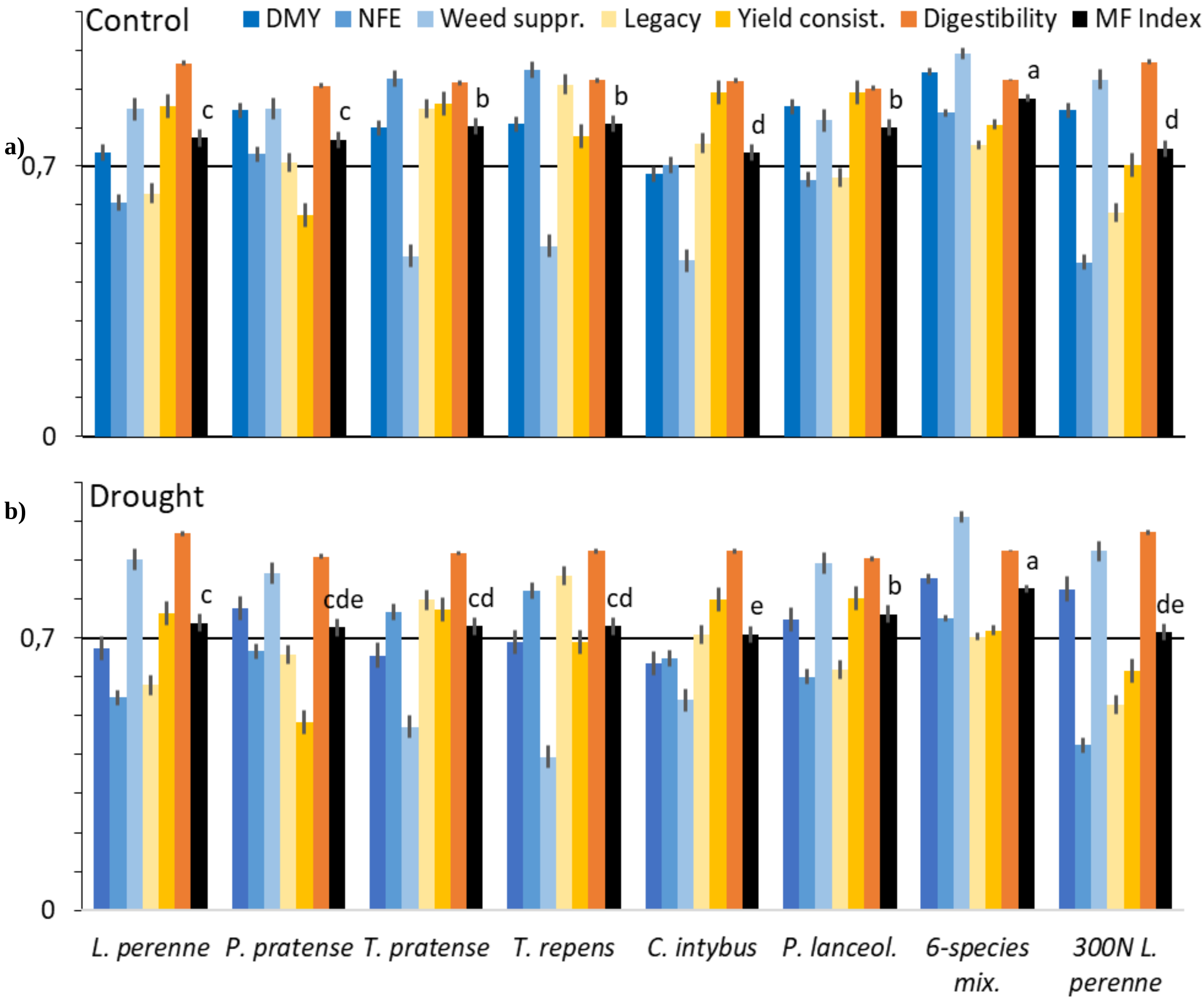

Figure S1. Modelled predictions (± s.e.) for performance of selected communities across multiple functions, for (a) rainfed control and (b) drought conditions. Each bar represents the standardized prediction for the corresponding function for all 150N monocultures, the six-species equi-proportional mixture (centroid) and the 300N *L. perenne* monoculture. The black bar shows the MF index, the unweighted average across all functions. Pairwise comparison tests were performed for the MF index (black bar) across the eight community types for rainfed and drought separately; within each group, bars that do not share a letter are significantly different (α = 0.05). The horizontal line displays a threshold of 70% of the best performing community.

Table S1. Variance-covariance matrix as estimated from the model described in eqn. 4.2. Fixed parameter estimates are in Table 1. An unstructured covariance structure is assumed for multivariate ecosystem functions from the same subplot, while a covariance is induced on individual ecosystem functions from two subplots within the same main plot to incorporate the split-plot design.

			Main plot											
			Sub plot						Sub plot					
			**DMY**	**Digest.**	**Legacy**	**NFE**	**Yield consist.**	**Weed suppr.**	**DMY**	**Digest.**	**Legacy**	**NFE**	**Yield consist.**	**Weed suppr.**
Main plot	Sub plot	**DMY**	0.00162	-0.00008	-0.00019	0.00073	-0.00157	0.00002	0	0	0	0	0	0
		**Digest.**	-0.00008	0.00020	0.00002	-0.00003	-0.00008	0.00002	0	0	0	0	0	0
		**Legacy**	-0.00019	0.00002	0.00442	0.00208	0.00051	-0.00009	0	0	0.00009	0	0	0
		**NFE**	0.00073	-0.00003	0.00208	0.00196	-0.00027	-0.00011	0	0	0	0.00022	0	0
		**Yield consist.**	-0.00157	-0.00008	0.00051	-0.00027	0.00696	-0.00002	0	0	0	0	0.00007	0
		**Weed suppr.**	0.00002	0.00002	-0.00009	-0.00011	-0.00002	0.00268	0	0	0	0	0	0.00107
	Sub plot	**DMY**	0	0	0	0	0	0	0.00162	-0.00008	-0.00019	0.00073	-0.00157	0.00002
		**Digest.**	0	0	0	0	0	0	-0.00008	0.00020	0.00002	-0.00003	-0.00008	0.00002
		**Legacy**	0	0	0.00009	0	0	0	-0.00019	0.00002	0.00442	0.00208	0.00051	-0.00009
		**NFE**	0	0	0	0.00022	0	0	0.00073	-0.00003	0.00208	0.00196	-0.00027	-0.00011
		**Yield consist.**	0	0	0	0	0.00007	0	-0.00157	-0.00008	0.00051	-0.00027	0.00696	-0.00002
		**Weed suppr.**	0	0	0	0	0	0.00107	0.00002	0.00002	-0.00009	-0.00011	-0.00002	0.00268

Table S2. Experimental design showing the plant diversity treatment for each community (each column). Shown for each community is: the number of functional groups (FG) and species sown, the proportion of each, the number of replicates and the fertiliser rate (1 = 150 kg N ha^{-1}, 2 = 300 kg N ha^{-1}). There were 43 main plots in total. Each main plot was divided into two sub-plots and the rainfed control or drought treatment imposed on one sub-plot (randomly selected).

	**Monocultures**							**Mixtures**												
**FG number**	1	1	1	1	1	1	1	1	1	1	2	2	2	3	3	3	3	3	3	3
**Species number**	1	1	1	1	1	1	1	2	2	2	4	4	4	5	5	5	5	5	5	6
**Functional group proportion**																				
-Grass	1	1	1					1			0.5	0.5		0.6	0.6	0.2	0.2	0.2	0.2	1/3
-Legume				1	1				1		0.5		0.5	0.2	0.2	0.6	0.6	0.2	0.2	1/3
-Herb						1	1			1		0.5	0.5	0.2	0.2	0.2	0.2	0.6	0.6	1/3
**Species proportion**																				
-*Lolium perenne*	1	1						0.5			0.25	0.25		0.6		0.1	0.1	0.1	0.1	1/6
-*Phleum pratense*			1					0.5			0.25	0.25			0.6	0.1	0.1	0.1	0.1	1/6
-*Trifolim pratense*				1					0.5		0.25		0.25	0.1	0.1	0.6		0.1	0.1	1/6
-*Trifolium repens*					1				0.5		0.25		0.25	0.1	0.1		0.6	0.1	0.1	1/6
-*Cichorium intybus*						1				0.5		0.25	0.25	0.1	0.1	0.1	0.1	0.6		1/6
-*Plantago lanceolata*							1			0.5		0.25	0.25	0.1	0.1	0.1	0.1		0.6	1/6
**Fertiliser application**	2	1	1	1	1	1	1	1	1	1	1	1	1	1	1	1	1	1	1	1
**Replicates**	4	3	3	3	3	3	3	2	2	2	2	2	2	1	1	1	1	1	1	3

a. yield

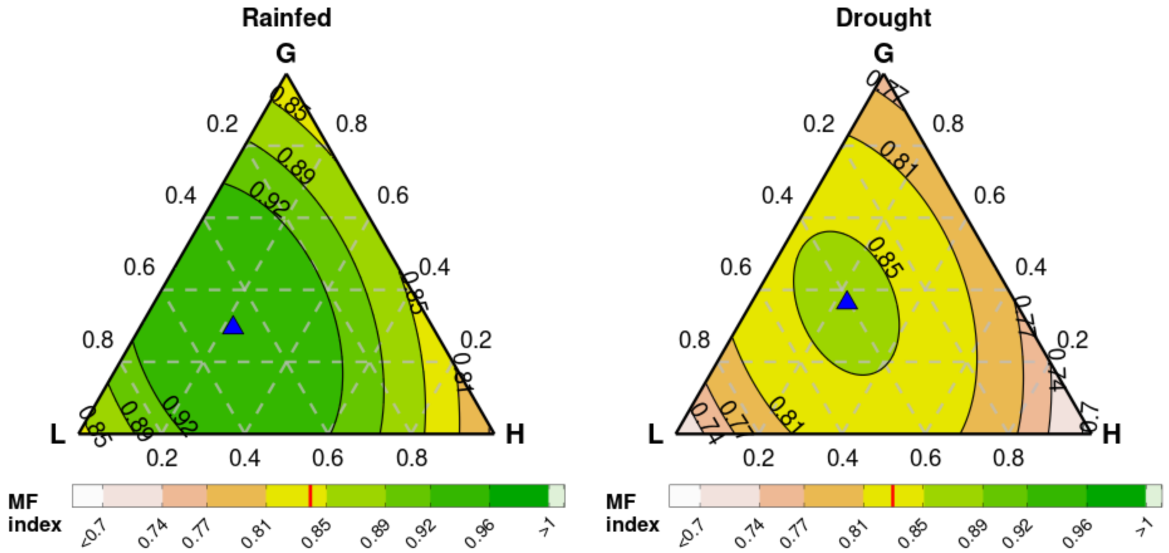

b. digestibility

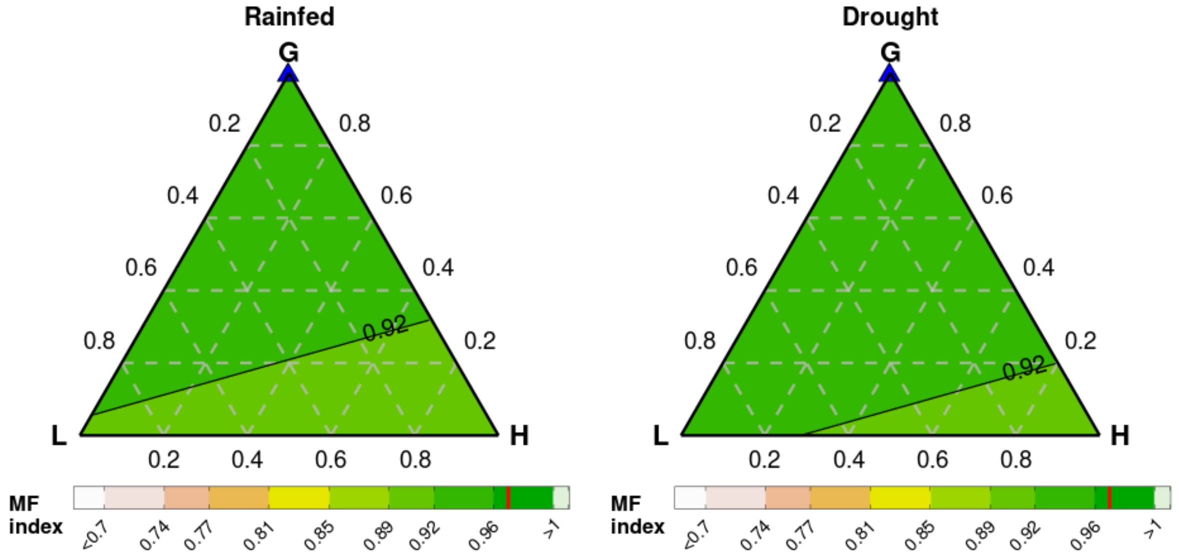

c. legacy effect

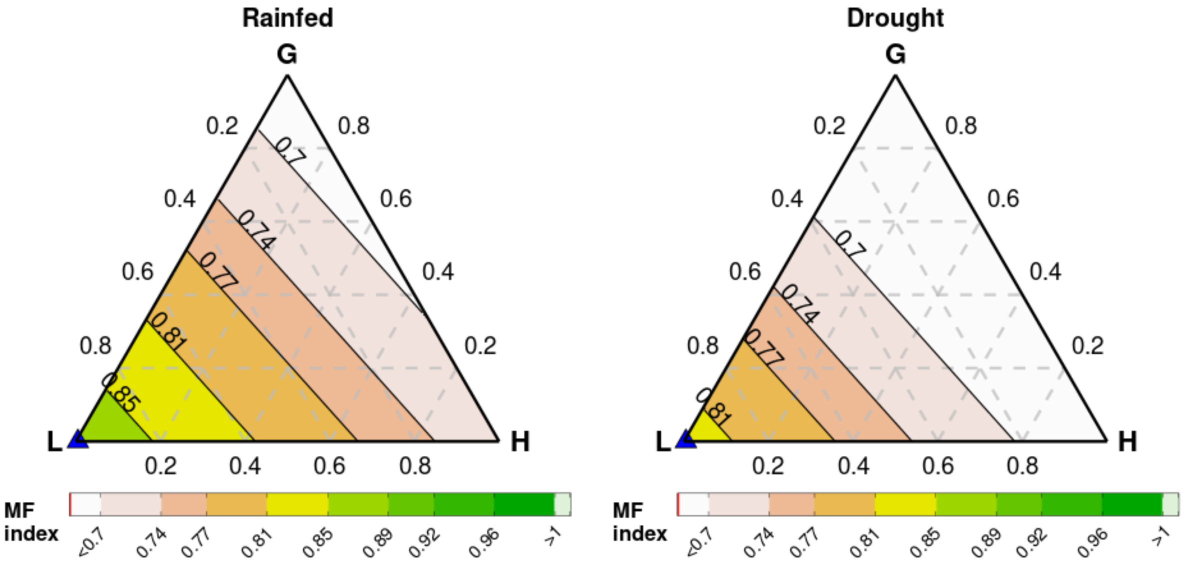

d. NFE

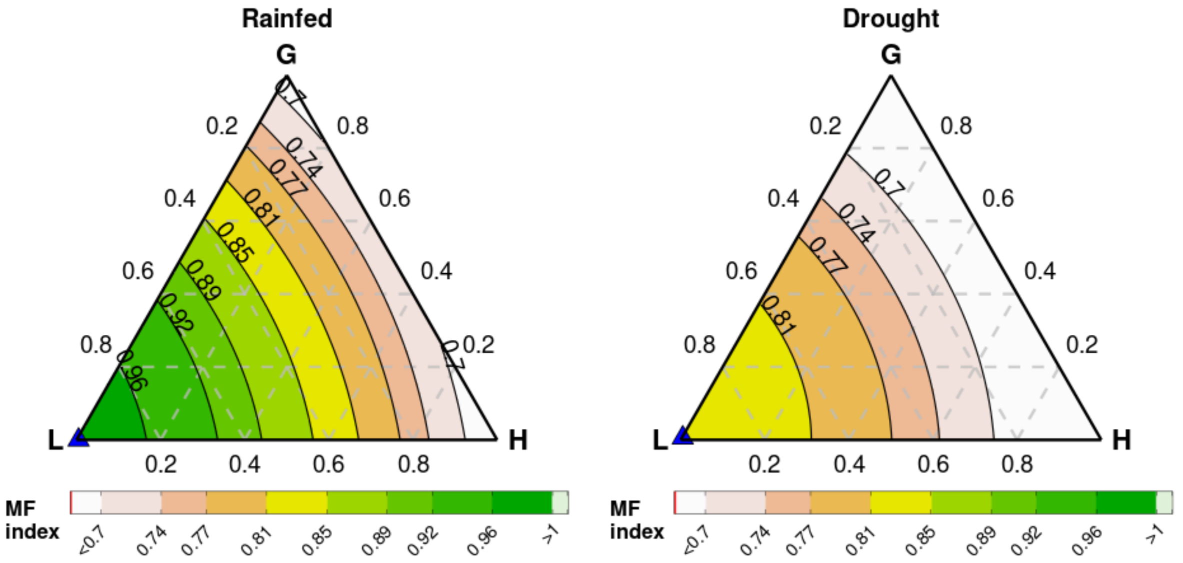

e. yield stability

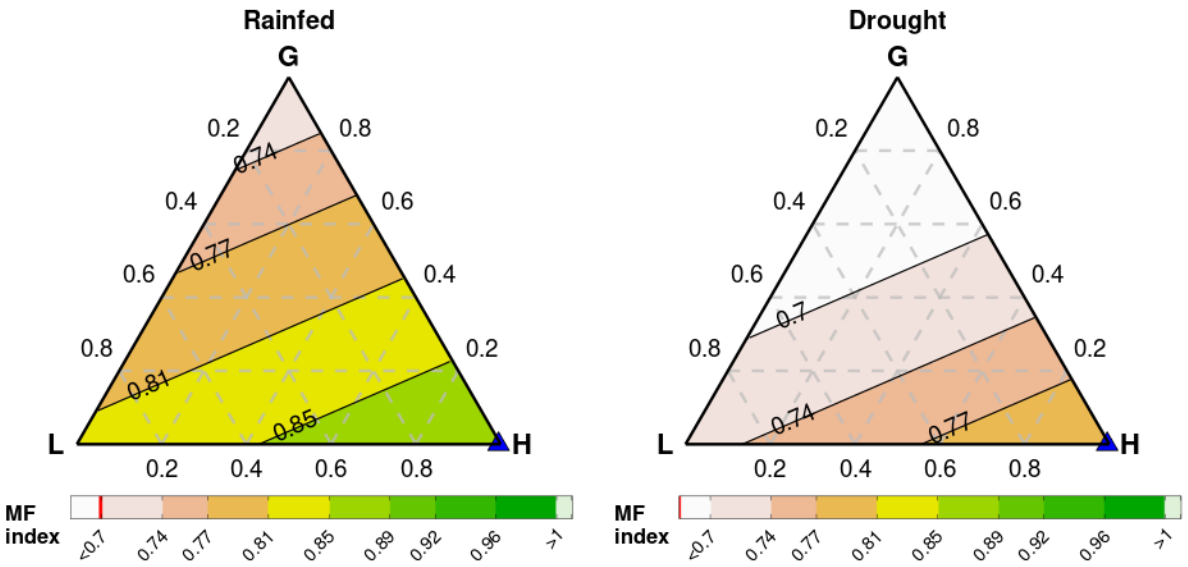

f. weed supression

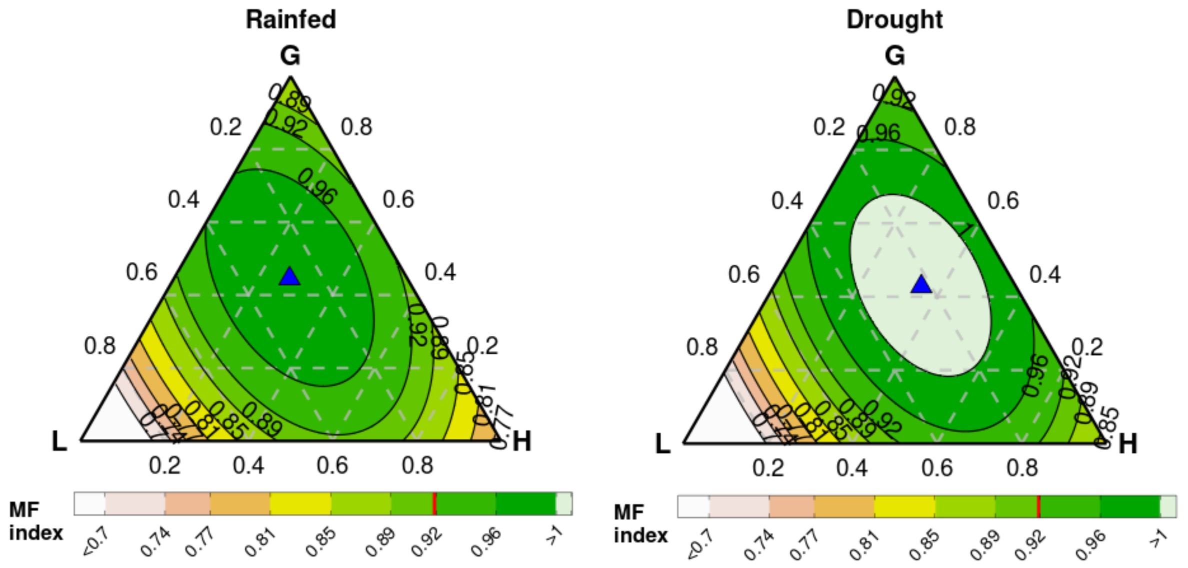

Figure S2. Ternary diagrams illustrating the relationship between diversity of the grass (G), herb (H) and legume (L) functional groups and each of the six individual functions (a to f), for both rainfed (left) and drought (right). The blue triangle indicates the best-performing community, while the red dash on the colour legend represents the performance of the 300N *Lolium perenne* monoculture. These ternary diagrams (and others) were produced using the shinyapp described in Box 1, by controlling the weighting sliders such that weights are 100% for each function in turn. Here, there are equal proportions for the two species from each functional group, but this can also be controlled in the shinyapp (Box 1).

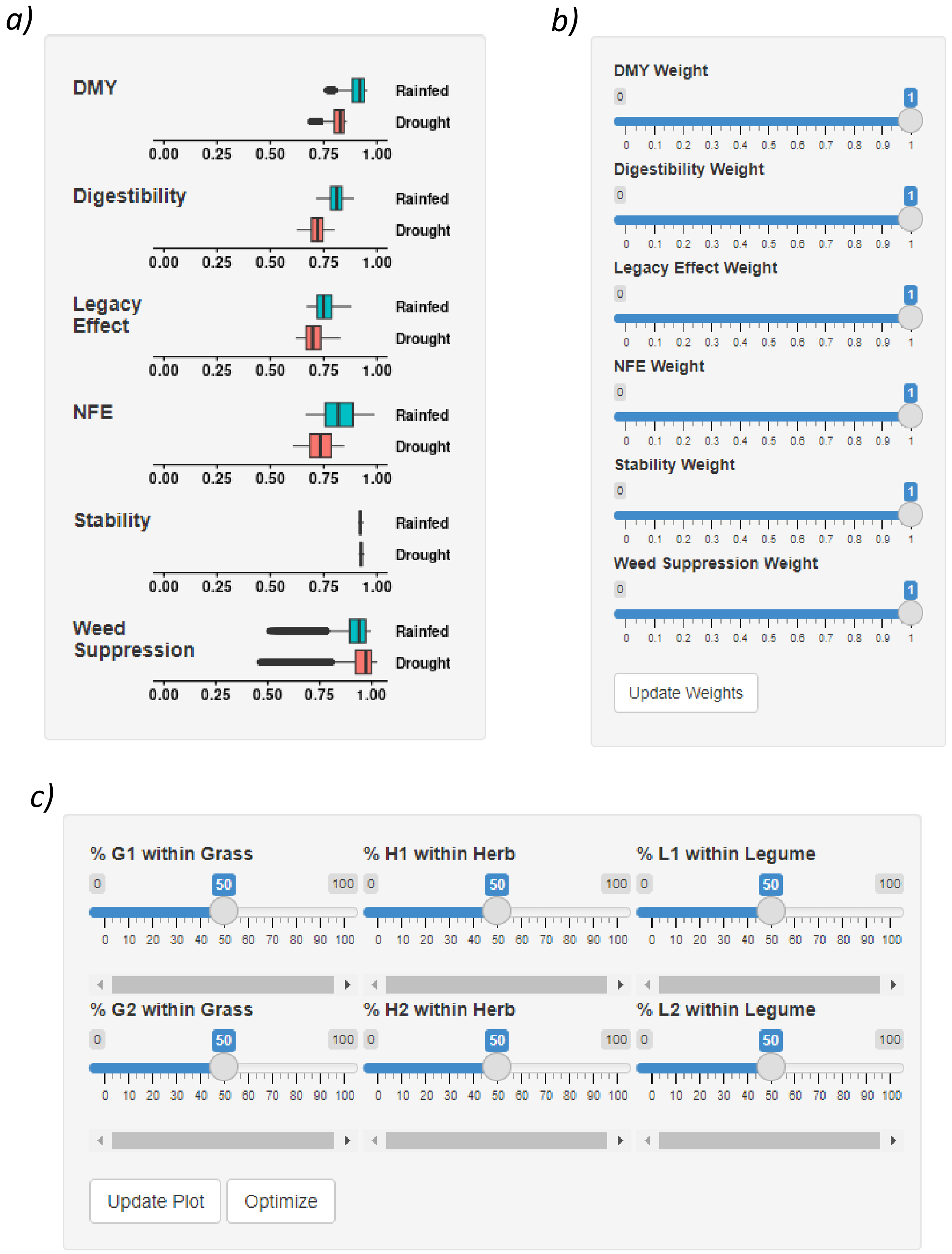

Figure S3. Control panels for the interactive visualisation described in Box 1. a) boxplots of individual functions across the drought and rainfed conditions, b) sliders for adjusting the weights of individuals functions when calculating the MF value and c) sliders for adjusting the relative proportion of the constituent species within a functional group. For example, sliding the

‘% G1 within Grass’ slider to 80% would cause the ‘% G2 within Grass’ slider to move to 20% and would imply that at every point in the functional group ternary space, the total grass proportion would consist of 80% of G1 and 20% of G2. Similar changes can be made for the other two functional groups such that one can construct a range of communities that comprise at least one grass, one legume and one herb species.
